# Loss of skeletal muscle mass affects the incidence of minimal hepatic encephalopathy: a case control study

**DOI:** 10.1186/s12876-020-01501-x

**Published:** 2020-11-09

**Authors:** Masakuni Tateyama, Hideaki Naoe, Motohiko Tanaka, Kentaro Tanaka, Satoshi Narahara, Takayuki Tokunaga, Takeshi Kawasaki, Yoko Yoshimaru, Katsuya Nagaoka, Takehisa Watanabe, Hiroko Setoyama, Yutaka Sasaki, Yasuhito Tanaka

**Affiliations:** 1 Department of Gastroenterology and Hepatology, Graduate School of Medical Sciences, Kumamoto University, Honjo 1-1-1, Chuo-ku, Kumamoto 860-8556 Japan; 2 Kumamoto Rosai Hospital, 1670 Takeharatyo, Yatsushiro City, Kumamoto Japan; 3 Department of Health and Nutrition, Nagasaki International University, 2825-7 Huis Ten Bosch Machi, Sasebo City, Nagasaki 859-3298 Japan

**Keywords:** Minimal hepatic encephalopathy, Skeletal muscle index, Liver cirrhosis, Sarcopenia

## Abstract

**Background:**

Sarcopenia is a syndrome characterized by progressive and systemic decreases in skeletal muscle mass and muscle strength. The influence or prognosis of various liver diseases in this condition have been widely investigated, but little is known about whether sarcopenia and/or muscle mass loss are related to minimal hepatic encephalopathy (MHE).

**Methods:**

To clarify the relationship between MHE and sarcopenia and/or muscle mass loss in patients with liver cirrhosis.

**Methods:**

Ninety-nine patients with liver cirrhosis were enrolled. MHE was diagnosed by a neuropsychiatric test. Skeletal mass index (SMI) and Psoas muscle index (PMI) were calculated by dividing skeletal muscle area and psoas muscle area at the third lumbar vertebra by the square of height in meters, respectively, to evaluate muscle volume.

**Results:**

This study enrolled 99 patients (61 males, 38 females). MHE was detected in 48 cases (48.5%) and sarcopenia in 6 cases (6.1%). Patients were divided into two groups, with or without MHE. Comparing groups, no significant differences were seen in serum ammonia concentration or rate of sarcopenia. SMI was smaller in patients with MHE (46.4 cm^2^/m^2^) than in those without (51.2 cm^2^/m^2^, *P* = 0.027). Similarly, PMI was smaller in patients with MHE (4.24 cm^2^/m^2^) than in those without (5.53 cm^2^/m^2^, *P* = 0.003). Skeletal muscle volume, which is represented by SMI or PMI was a predictive factor related to MHE (SMI ≥ 50 cm^2^/m^2^; odds ratio 0.300, *P* = 0.002, PMI ≥ 4.3 cm^2^/m^2^; odds ratio 0.192, *P* = 0.001).

**Conclusions:**

Muscle mass loss was related to minimal hepatic encephalopathy, although sarcopenia was not. Measurement of muscle mass loss might be useful to predict MHE.

## Background

Hepatic encephalopathy (HE) is a complication of liver cirrhosis, and reduces quality of life (QOL) for the patient. Minimal HE (MHE) is a neuropsychiatric abnormality without clinical overt symptoms of HE [1–3], and is diagnosed using sensitive neuropsychological and neurophysiological examinations. Difficulty in diagnosing MHE aggravates not only QOL, but also prognosis for patients [4–6]. To improve these outcomes, early detection of MHE is needed.

Ammonia is a typical molecule inducing encephalopathy. In cirrhotic patients, ammonia metabolism is attenuated in the liver. On the other hand, muscle plays an important role in ammonia decomposition. Loss of muscle mass would thus presumably induce deteriorations in ammonia metabolism. In addition, ammonia itself reportedly inhibits protein synthesis in muscle. Sarcopenia is thus an unfavorable condition in terms of ammonia metabolism, since it involves losses of both mass and strength in skeletal muscle. Furthermore, sarcopenia affects clinical outcomes in various liver diseases [7]. The frequency of sarcopenia increases with the progression of liver disease.

Although HE is associated with sarcopenia and muscle mass loss, whether MHE is associated with sarcopenia and/or muscle mass loss remains unclear. The purpose of this study was to clarify the relationship between MHE and sarcopenia and muscle mass loss in patients with liver cirrhosis.

## Methods

### Study cohort

Patients with cirrhosis were enrolled from May 2017 to December 2018 in Kumamoto University Hospital. Clinical records, laboratory data and clinical imaging findings were collected at the time of entry. Patients with cirrhosis were diagnosed by clinical findings and/or pathological results. Exclusion criteria were as follows: (1) history of overt HE; (2) patients with cognitive impairment (e.g., Alzheimer disease); (3) presence of advanced Hepatocellular carcinoma (HCC) on admission (although HCC patients who met Milan criteria were not excluded: single tumor < 5 cm in maximum diameter or ≤ 3 tumors, all < 3 cm in diameter [8]); or (4) use of psychiatric drugs (e.g., anti-Parkinson’s disease agents, antipsychotics, or antidepressants) or narcotics.

### Testing for MHE

To diagnose MHE, we used the neuropsychiatric-test (NP-test) application on a tablet computer (iPad; Apple, USA). This application was provided by Otsuka Pharmaceutical Co. Ltd. (Japan) on the site of the Japan Society of Hepatology. We used the following four tests: number connection test (NCT)-A; NCT-B; the digit symbol test; and the block design test. This application includes these tests to diagnose MHE. If abnormal results are obtained on two or more of these tests, the patient was diagnosed with MHE [1].

### Judgement of sarcopenia

**Fig. 1:**
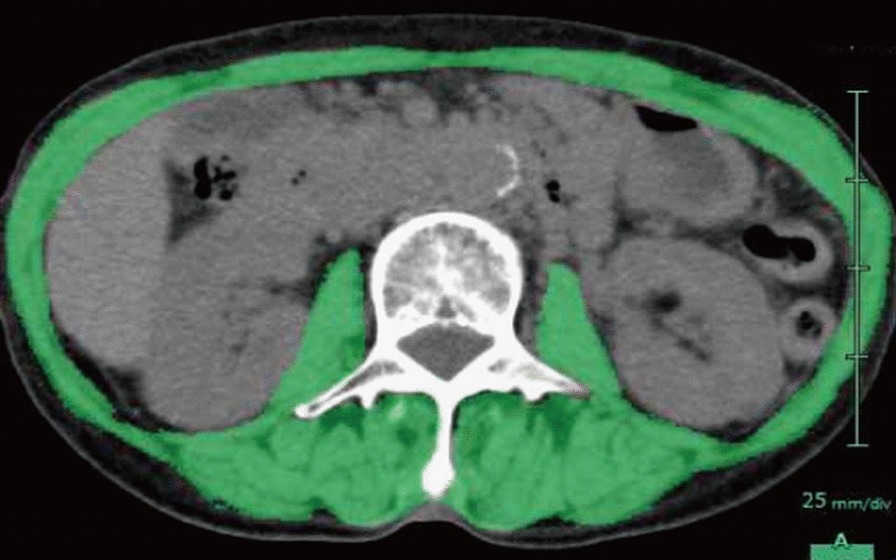
Representative of CT scan image. After skeletal muscle area was estimated by subtracting the areas of visceral fat and subcutaneous fat from the total area, it was determined on CT by manual tracing (green area). The areas of visceral and subcutaneous fat were calculated by the abdominal application software, automatically

To diagnose sarcopenia, we used the criteria for sarcopenia proposed by the Japan Society of Hepatology [7]. The criteria comprise grip strength and muscle mass. Grip strength was measured using a Smedley-type dynamometer and the mean of two measurements for both sides was used as the measured value. Skeletal muscle area was determined on CT at the level of the third lumbar vertebra (L3) on a slice showing both transverse processes and was measured by manual tracing using the SYNAPSE VINCENT 3D image analysis system (Fujifilm, Japan) (Fig. 1). Simultaneously, the area of both left and right iliopsoas muscle was measured by manual tracing as psoas muscle area. Skeletal muscle index (SMI) was calculated as follow: skeletal muscle area at the L3 level was divided by the square of height in meters (muscle mass area [cm^2^]/height^2^[m^2^]). Likewise, psoas muscle index (PMI) was calculated by dividing psoas muscle area at the L3 level by the square of height in meters (muscle mass area [cm^2^]/height^2^[m^2^]) [9]. Cut-off values for grip strength (26 kg in men, 18 kg in women) and SMI (42 cm^2^/m^2^ in men; 38 cm^2^/m^2^ in women) were based on the guidelines for sarcopenia in liver disease issued by the Japan Society of Hepatology [7]. If the patient showed values below cut-off levels in both grip strength and SMI, sarcopenia was diagnosed. We also determined muscle strength loss if the value was below the cut-off level for grip strength, and muscle mass loss if the value was below the cut-off level for skeletal muscle index according to sex.

### Statistical analysis

To explore differences between groups, Student’s t test or the non-parametric test was used for continuous variables, and the chi-squared test was used for categorical variables. Continuous variables are expressed as median (range) and categorical variables as number (percentage). Continuous variables were dichotomized with respect to the median value or clinically meaningful values validated by receiver operating characteristic analysis in logistic regression analyses. The cut-off values of these variables, calculated by CT image such as SMI, were determined by ROC curve and youden index. Logistic regression analysis was performed to examine predictors of MHE. Values of *P* < 0.05 were considered statistically significant. Data analysis was performed using SPSS version 22 statistical software (IBM SPSS Japan, Tokyo, Japan).

## Results

### Patient characteristics

Background characteristics of patients are shown in Table 1. Of the 99 patients, median age was 70.0 years old and 61 patients (61.6%) were men. Etiology was hepatitis B virus (HBV) in 18 patients, hepatitis C virus (HCV) in 52 patients, and neither HBV nor HCV in 29 patients. Thirty HCV-infected patients achieved SVR among 52 patients. Among all patients, 29 (29.3%) did not have HCC, while 58 (58.6%) showed complications of gastroesophageal varices. Sixty patients were classified as Child–Pugh class A. For all patients, median grip strength was 24.6 kg and median SMI was 47.9 cm^2^/m^2^.

**Table 1:** Patient background characteristics (n = 99)

Age (years)	70.0 (42–90)
Male, n (%)	61 (61.6)
Etiology	
HBV, n (%)	18 (18.2)
HCV (including SVR), n (%)	52 (52.5)
NBNC, n (%)	29 (29.3)
Taking BCAA, n (%)	28 (28.3)
Taking diuretic, n (%)	25 (25.3)
Alcohol, n (%)	39 (39.4)
Diabetes, n (%)	33 (33.3)
HCC, n (%)	70 (70.7)
Esophageal or gastric varices, n (%)	40 (40.4)
Porto-systemic shunts, n (%)	35 (35)
Ascites, n (%)	10 (10.1)
Child–Pugh Class	
A	60 (60.6)
B	25 (25.3)
C	4 (4.0)
Sarcopenia, n (%)	6 (6.1)
Body height (m)	1.59 (1.37–1.90)
Body weight (kg)	57.4 (36.6–92.8)
BMI (kg/m^2^)	23.7 (16.0–33.9)
Grip strength (kg)	24.6 (7.2–43.5)
Hemoglobin (g/L)	128 (79–169)
Platelets (× 10^3^/μL)	107 (21–390)
ALT (U/L)	23 (8–123)
γ-GTP (U/L)	41 (13–696)
Cholinesterase (U/L)	206 (53–379)
Albumin (g/L)	38 (22–49)
Total cholesterol (mmol/L)	4.16 (2.20–6.59)
Ammonia (mol/L)	31.1 (4.11–157.37)
Prothrombin time (%)	80 (31–115)
Sodium (mEq/L)	140 (133–146)
BCAA (mmol/L)	423.7 (228.3–1459.9)
Tyrosine (mmol/L)	96.2 (37.1–174.0)
BTR	4.75 (1.44–11.07)
Subcutaneous fat area (cm^2^)	101.61 (3.84–332.49)
Visceral fat area (cm^2^)	111.37 (7.03–287.84)
CT level of skeletal muscles (HU)	22.70 (− 16.42–47.52)
Skeletal muscle area (cm^2^)	118.74 (72.12–188.46)
Skeletal muscle index (cm^2^/m^2^)	47.9 (29.4–68.5)
Psoas muscle area (cm^2^)	12.47 (5.11–24.49)
Psoas muscle index (cm^2^/m^2^)	5.02 (2.45–9.20)

Continuous variables are expressed as medians. Numbers in parentheses of continuous variables show each range of variables

HBV, hepatitis B virus; HCV, hepatitis C virus; NBNC, non-B, non-C etiology; HCC, hepatocellular carcinoma; BMI, body mass index; ALT, alanine aminotransferase; γ-GTP, γ-glutamyl transpeptidase; CRP, C-reactive protein; BCAA, branched-chain amino acids; BTR, molar ratio of branched-chain amino acids to tyrosine; CT, computed tomography

### Difference between presence or absence of MHE

Table 2 shows a comparison of patient background characteristics between patients with and without MHE. Forty-eight patients were judged as positive for MHE. Body weight was lower in patients with MHE than in those without MHE. Concentrations of ammonia were similar between groups. In amino acid analysis (concentration of branched-chain amino acids (BCAA), that of Tyrosine and molar ratio of BCAA to tyrosine (BTR)), no differences were evident between groups. On the other hand, grip strength, body height, body weight, body mass index (BMI), skeletal muscle area at the L3 level, SMI, psoas muscle area and PMI differed significantly between groups. Median SMI in patients with and without MHE were 46.4 cm^2^/m^2^ and 51.2 cm^2^/m^2^, respectively.

**Table 2:** Comparison of patient background characteristics between presence or absence of minimal hepatic encephalopathy

	MHE (-) (n = 51)	MHE ( +) (n = 48)	*P *value
Age (years)	72	68	0.944
Male, n (%)	34 (66.7)	27 (56.3)	0.287
Etiology			0.677
HBV, n (%)	11 (21.6)	7 (14.6)	
HCV (include SVR), n (%)	24 (47.0)	28 (58.3)	
NBNC, n (%)	16 (31.4)	13 (27.1)	
Taking BCAA, n (%)	11 (21.6)	17 (35.4)	0.126
Taking diuretic, n (%)	12 (23.5)	13 (27.1)	0.684
Alcohol, n (%)	22 (43.1)	17 (35.4)	0.432
Diabetes, n (%)	17 (33.3)	16 (33.3)	1.000
HCC, n (%)	36 (70.6)	34 (75.0)	0.979
Esophageal or gastric varices, n (%)	19 (38.0)	21 (43.8)	0.563
Porto-systemic shunts, n (%)	22 (43.1)	13 (27.1)	0.095
Ascites, n (%)	4 (7.8)	6 (12.5)	0.332
Child–Pugh Class			0.879
A	30 (66.7)	30 (68.4)	
B or C	15 (33.3)	14 (31.8)	
Sarcopenia, n (%)	3 (5.9)	3 (6.3)	0.632
Body height (m)	1.61	1.56	0.023
Body weight (kg)	60.8	53.9	0.002
BMI (kg/m^2^)	24.52	22.66	0.021
Grip strength (kg)	28.3	21.9	0.041
Hemoglobin (g/L)	133	124	0.181
Platelet (× 10^3^/μL)	107	106.5	0.563
ALT (U/L)	26	23	0.385
γ-GTP (U/L)	43	39	0.975
Cholinesterase (U/L)	210	203	0.416
Albumin (g/L)	39	38	0.863
Total cholesterol (mmol/L)	4.16	4.16	0.991
Ammonia (μmol/L)	31.1	30.5	0.674
Prothrombin time (%)	79.0	81.5	0.483
Sodium (mEq/L)	140	139.5	0.946
BCAA (mmol/L)	465.9	404.0	0.093
Tyrosine (mmol/L)	99.5	91.1	0.076
BTR	4.62	4.78	0.978
Subcutaneous fat area (cm^2^)	119.99	97.35	0.198
Visceral fat area (cm^2^)	111.37	111.09	0.385
CT level of skeletal muscle (HU)	23.46	22.51	0.287
Skeletal muscle area (cm^2^)	125.85	108.23	0.003
Skeletal muscle index (cm^2^/m^2^)	51.22	46.40	0.027
Psoas muscle area (cm^2^)	14.34	10.85	0.002
Psoas muscle index (cm^2^/m^2^)	5.53	4.24	0.003

Continuous variables are expressed as medians

SMI, skeletal muscle index; PMI, psoas muscle index

### Sarcopenia and muscle mass loss

**Fig. 2:**
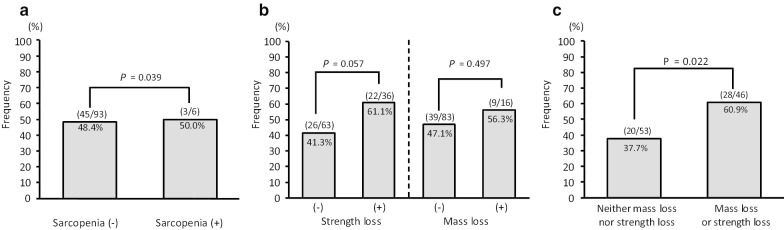
Incidence of MHE with and without sarcopenia (**a**), muscle strength loss or mass loss (**b**, **c**). In cases with strength loss or volume loss, the incidence of MHE was similar (**b**). But, incidence of MHE in patients with volume loss or strength loss was significantly higher than that in patients with neither volume loss nor strength loss (**c**)

In this cohort, 6 patients (6.7%) were diagnosed with sarcopenia. Figure 2 shows the incidence of MHE stratified by the presence of sarcopenia. Incidence of MHE in patients with and without sarcopenia was 50.0% and 48.4%, respectively (Fig. 2a). No significant difference in the incidence of MHE was evident regardless of muscle strength loss or muscle mass loss (Fig. 2b). However, we found that presence of either muscle mass loss or strength loss was significantly associated with higher incidence of MHE, whereas absence of either was not (Fig. 2c).

### Predictive factors for incidence of MHE

**Fig. 3:**
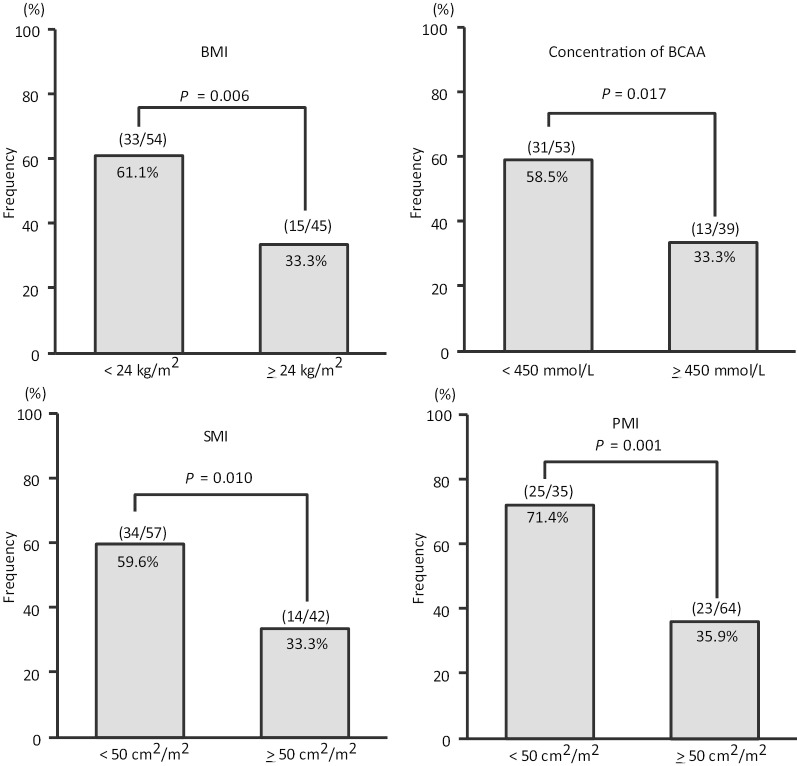
Incidence of MHE by BMI, BCAA concentration, SMI and PMI. Incidence of minimal hepatic encephalopathy was significantly higher in cases below each cut-off level than in cases above each cut-off

Next, we performed univariate analysis to elucidate factors associated with MHE using all variables in Table 2. Cut-off values of SMI and PMI defined by ROC curve were 50 cm^2^/m^2^ and 4.3 cm^2^/m^2^, respectively. Table 3 showed only the variables with statistically significant　differences except for those that were not significantly different by univariate analysis. Body weight, BMI, concentration of BCAA, skeletal muscle area, SMI, psoas muscle area and PMI were significantly correlated with the presence of MHE (Table 3), whereas sarcopenia was not significantly associated with MHE. Since BMI and SMI include body weight and skeletal muscle area, respectively, we focused on BMI, concentration of BCAA and SMI, and performed multivariate analysis using these. As a result, SMI was detected as the only significant factor (≥ 50 cm^2^/m^2^, odds ratio (OR) 0.300, *P* = 0.006). Similarly, when using PMI instead of SMI, PMI was detected as the only significant factor (≥ 4.3 cm^2^/m^2^, odds ratio (OR) 0.192, *P* = 0.001) (Table 3). The incidence rate of MHE was 59.6% in patients with SMI < 50 cm^2^/m^2^, and 33.3% in patients with SMI ≥ 50 cm^2^/m^2^ and that was 71.4% in patients with PMI < 4.3 cm^2^/m^2^, and 35.9% in patients with PMI ≥ 4.3 cm^2^/m^2^, respectively (Fig. 3). In addition, the all six patients with sarcopenia belonged to the low BMI group, which was less than 24 kg/m^2^.

**Table 3:** Predictive factors for the presence of minimal hepatic encephalopathy

	Univariate	
	Odds ratio	*P* value
Body weight, kg		
< 60	1	
≥ 60	0.357	0.016
BMI, kg/m^2^		
≥ 24	1	
≥ 24	0.318	0.007
BCAA, μmol/L		
< 450	1	
≥ 450	0.355	0.018
Skeletal muscle area, cm^2^		
< 120	1	
≥ 120	0.297	0.004
Skeletal muscle index, cm^2^/m^2^		
< 50	1	
≥ 50	0.338	0.011
Psoas muscle area, cm^2^		
< 9.5	1	
≥ 9.5	0.205	0.002
Psoas muscle index, cm^2^/m^2^		
< 4.3	1	
≥ 4.3	0.224	0.001

	Multivariate (using SMI)		
	Odds ratio	*P *value	95% CI
BMI, kg/m^2^			
< 24			
≥ 24			
BCAA, μmol/L			
< 450			
≥ 450			
Skeletal muscle index, cm^2^/m^2^			
< 50			
≥ 50	0.300	0.006	0.126–0.712

	Multivariate (using PMI)		
	Odds ratio	*P* value	95% CI
BMI, kg/m^2^			
< 24			
≥ 24			
BCAA, μmol/L			
< 450			
≥ 450			
Psoas muscle index, cm^2^/m^2^			
< 4.3			
≥ 4.3	0.192	0.001	0.075–0.491

CI, confidence interval

### Incidence of MHE classified by sex and age

**Fig. 4:**
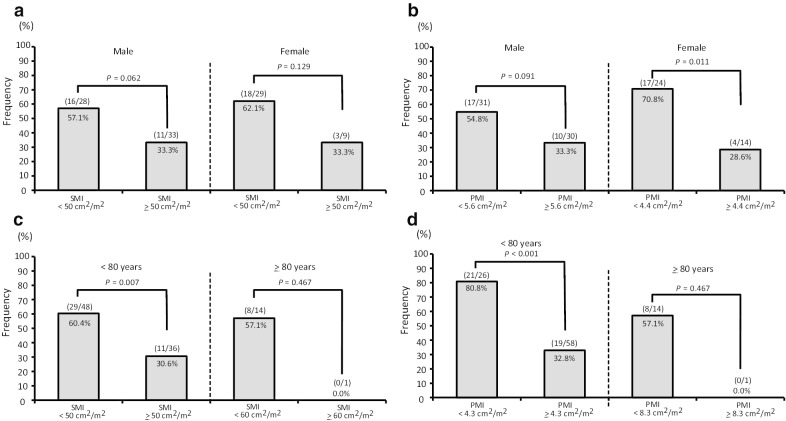
Incidence of minimal MHE by sex (**a**, **b**) and age (**c**, **d**). In cases with lower SMI or PMI, incidence of minimal hepatic encephalopathy tended to be higher than in cases above the cut-off, although the difference was not significant (**a**, **b**). In patients less than 80 years old, incidence of minimal hepatic encephalopathy was significantly higher in cases with lower SMI or PMI than in cases with them above the cut-off. In patients more than 80 years old, no difference was seen between below and above the cut-off (**c**,** d**)

Since SMI varies among sex, we examined SMI and the incidence of MHE by sex. Median SMI was 51.0 cm^2^/m^2^ in males and 45.1 cm^2^/m^2^ in females (Table 4). The incidence of MHE was higher in the group with SMI below the cut-off level than in the group with SMI above the cut-off in both males and females (Fig. 4a). Regarding the frequency of MHE, there was a similar tendency in PMI (Fig. 4b). Next, patients with high age showed the possibility of declines in higher brain functions such as recognition and decision making. We also examined the incidence of MHE by age. Table 4 shows median SMI classified by the age threshold of 80 years old. While grip strength differed between subjects under or over 80 years old, SMI and PMI were similar between groups. The incidence of MHE differed significantly between groups classified using the SMI or PMI level below and above the cut-off level in patients < 80 years old (Fig. 4c, d).

**Table 4:** SMI and PMI by sex and age

	Male (n = 61)	Female (n = 38)	*P *value
(A) SMI and PMI by sex			
Grip strength (kg)	30.9 (14.2–43.5)	17.6 (7.2–25.2)	< 0.001
Skeletal muscle area (cm^2^)	131.90 (86.99–188.46)	98.32 (72.12–143.99)	< 0.001
Skeletal muscle index (cm^2^/m^2^)	51.0 (29.4–68.5)	45.1 (32.5–65.0)	0.001
Psoas muscle area (cm^2^)	14.88 (6.46–24.49)	8.89 (5.11–15.79)	< 0.001
Psoas muscle index (cm^2^/m^2^)	5.6 (3.1–9.2)	4.0 (2.5–7.6)	< 0.001

	< 80 years (n = 84)	≥ 80 years (n = 15)	*P *value
(B) SMI and PMI by age			
Grip strength (kg)	26.8 (8.9–43.5)	17.0 (7.2–28.3)	< 0.001
Skeletal muscle area (cm^2^)	122.01 (72.12–188.46)	99.59 (72.31–173.66)	0.023
Skeletal muscle index (cm^2^/m^2^)	47.9 (29.4–68.5)	47.3 (32.5–66.7)	0.507
Psoas muscle area (cm^2^)	13.19 (5.11–24.49)	9.00 (5.33–23.29)	0.028
Psoas muscle index (cm^2^/m^2^)	5.2 (2.5–9.2)	4.1 (2.5–8.9)	0.212

SMI, Skeletal muscle index; PMI, Psoas muscle index

## Discussion

This study showed that MHE was affected by skeletal muscle mass and suggests that skeletal muscle may play an important role in MHE. As a neuropsychiatric abnormality without overt clinical symptoms of HE [1–3], MHE is considered to be the primary status of overt HE [10]. MHE leads to deteriorations in QOL [4, 5] and impairment of driving skill for motor vehicles [11–17]. Moreover, MHE is associated with falls [18, 19], learning impairment [20] and poor prognosis [6, 19, 21]. In addition, MHE is reportedly related to the survival of patients with liver cirrhosis [6, 22]. Early detection of patients with MHE is therefore necessary.

In patients with cirrhosis, ammonia, as the main substance causing HE, is increased in serum, since its metabolism is attenuated in the liver. Ammonia metabolism by skeletal muscle can compensate for impaired hepatic metabolism [23–25]. Moreover, loss of skeletal muscle readily occurs in patients with cirrhosis due to alterations in protein turnover, energy disposal or metabolism [26]. Generally, skeletal muscle loss exacerbates deficiencies in detoxification of ammonia, inducing HE. Qureshi et al. reported that ammonia level correlates with the severity of HE [27]. Although the concentration of ammonia in our study showed no significant difference between patients with or without MHE, loss of skeletal muscle mass may affect the presence of MHE in patients with liver cirrhosis. In addition, Qiu et al. reported that hyperammonemia led to inhibition of protein synthesis through the increase in myostatin mediated by nuclear factor-κB [28]. These findings suggest that hyperammonemia and skeletal muscle loss might affect each other in a bi-directional manner.

Sarcopenia or muscle loss is considered a predictor of impaired QOL [29], postoperative major complication [30], prognosis [31–34] and physical disability [35]. Since advanced-stage HCC influences nutritional status, we excluded patients with HCC who did not meet Milan criteria [8] from the present study. Hanai et al. described sarcopenia as a predictor of MHE [36]. Moreover, several reports have shown that sarcopenia or muscle depletion affects the risk of overt HE or MHE [37–39]. These reports potentially support our result regarding patients without advanced HCC. According to the guidelines for sarcopenia in liver disease issued by the Japan Society of Hepatology, the proportion of sarcopenia in patients with cirrhosis varies within the range of 10–70% [7]. Because too few patients with sarcopenia were enrolled in our study, sarcopenia might not have been detected as a predictor of MHE. The exclusion criteria for advanced HCC in our study might have caused the discrepancy between our study and previous reports [36, 37]. With regards to cirrhotic patients without HCC or with HCC within Milan criteria, although the incidence of sarcopenia was quite low, we could predict the incidence of MHE in terms of muscle mass loss. We showed that PMI, as well as SMI were related to the presence of MHE. Since the measurement of psoas muscle area is easier than that of skeletal muscle area in the clinical setting, it can be also useful to check PMI as an indicator of muscle volume.

Skeletal muscle, SMI, skeletal muscle area and PMI were significant different between male and female. We examined the association between MHE and SMI or PMI separately for men and women, but the frequency of MHE was higher in the group with SMI or PMI below the cut-off level than in the group with them above it. This result may be due to a gender-independent relationship between muscle volume and MHE. Mild cognitive impairment (MCI) was not completely excluded because of the difficulty detecting this pathology in clinical settings. To remove this bias, we additionally analyzed the incidence of MHE among subjects under 80 years old, to reduce the possibility of MCI. The incidence of MHE differed significantly between cases below and above the cut-off level of SMI in patients under 80 years old.

Although various drugs have been given for patients with MHE [40–44] to decrease ammonia levels or improve mental status, drugs that are effective against MHE are unclear. We showed that the concentration of BCAA correlated with the presence of MHE, suggesting that administration of BCAA may be a reasonable treatment for MHE, because of its potential efficacy ameliorating skeletal muscle mass loss. Since current study was a case–control study, which fixed observation point, we were not able to evaluate whether the administration of BCAA would effect on MHE. Further study is needed to evaluate the curative or preventive effect of BCAA on MHE.

Several limitations to this study must be considered. First, the number of patients enrolled in this study was small. It may be too small to decide the appropriate cut-off level using ROC curve. Further analysis of a larger number of patients is needed to confirm the current findings and suitable cut-off value of SMI and PMI. Second, we used skeletal muscle area at the L3 level for the evaluation of muscle mass. Although this SMI was previously reported to correlate with SMI as calculated using bioelectrical impedance analysis (BIA) [7], we did not evaluate skeletal muscle mass by BIA. Third, we used the NP-test to diagnose MHE. The Psychometric Hepatic Encephalopathy Score (PHES), Inhibitory Control Test (ICT), electroencephalography, critical flicker frequency, and Stroop EncephAlapp are also available to diagnose MHE, and the combination of these tests is reportedly more useful than single tests [2, 45] Although we used NP-tests as a simple method in the clinical setting, how many and which types of tests are best and are most reliable for diagnosing MHE remains unclear.

## Conclusions

In conclusion, the present findings suggest that muscle mass loss might serve as a predictive factor for MHE among patients with liver cirrhosis and without advanced HCC. The therapeutic strategy for muscle mass loss would contribute to preventing occurrence of MHE.

## Data Availability

The datasets used and/or analyzed during the current study are available from the corresponding author on reasonable request.

## Abbreviations

HE: Hepatic encephalopathy
QOL: Quality of life
MHE: Minimal hepatic encephalopathy
HCC: Hepatocellular carcinoma
NP-test: The neuropsychiatric-test, NCT-A: number connection test-A
NCT-B: Number connection test-B
SMI: Skeletal muscle index
PMI: Psoas muscle index
HBV: Hepatitis B virus
HCV: Hepatitis C virus
BCAA: Branched-chain amino acids
BTR: Tyrosine and molar ratio of BCAA to tyrosine
BMI: Body mass index
MCI: Mild cognitive impairment
BIA: Bioelectrical impedance analysis
PHES: Psychometric Hepatic Encephalopathy Score
ICT: Inhibitory control test
